# Extended-spectrum beta-lactamase-producing *Enterobacteriaceae (*ESBL-PE) among travellers to Africa: destination-specific data pooled from three European prospective studies

**DOI:** 10.1186/s12879-018-3245-z

**Published:** 2018-07-23

**Authors:** Tinja Lääveri, Jessica A. Vlot, Alje P. van Dam, Hanni K. Häkkinen, Gerard J. B. Sonder, Leo G. Visser, Anu Kantele

**Affiliations:** 1 Inflammation Center, Division of Infectious Diseases, University of Helsinki and Helsinki University Hospital, POB 348, FIN-00029 HUS, Helsinki, Finland; 2 Department of Infectious Diseases, Leiden University Medical Center, Leiden, The Netherlands; 3 Department of Infectious Diseases, Public Health Service (GGD), Amsterdam, The Netherlands; 4 Department of Internal Medicine, Division of Infectious Diseases, Tropical Medicine and AIDS, Academic Medical Centre, Amsterdam, The Netherlands; 5 Clinicum, University of Helsinki, Helsinki, Finland; 6 Aava Travel Clinic, Medical Centre Aava, Helsinki, Finland; 7 Unit of Infectious Diseases, Karolinska Institutet, Solna, Stockholm Sweden

**Keywords:** ESBL, MDR, Extended-spectrum beta-lactamase, *Enterobacteriaceae*, Africa, Antimicrobials, Travel

## Abstract

**Background:**

One third of travellers to low- and middle-income regions of the tropics and subtropics become colonized by extended-spectrum beta-lactamase-producing *Enterobacteriaceae* (ESBL-PE). The risk varies by destination and, for each traveller, may be substantially further increased by travellers’ diarrhoea (TD) and antibiotic use. Despite the risk of TD in Africa, ESBL-PE acquisition rates in all studies are lower there than in Asia. Africa has become increasingly popular as a destination for international travellers, yet minimal data are available from the continent’s subregions and countries.

**Methods:**

We analysed subregion- and country-specific data on carriage and risk factors for ESBL-PE colonization pooled from three prospective studies conducted between 2009 and 2013 among Finnish and Dutch travellers. The data were subjected to multivariable analysis of risk factors. In addition, we compared our data to two recent large investigations reporting data by subregion and country.

**Results:**

Our joint analysis comprised data on 396 travellers. The ESBL-PE colonization rate was highest in Northern Africa, followed by Middle and Eastern Africa, and lowest in Southern and Western Africa. Of individual countries with more than 15 visitors, the highest rates were seen for Egypt (12/17; 70.6%), Ghana (6/23; 26.1%), and Tanzania (14/81; 17.3%); the rates among travellers to Egypt were comparable to those reported in South and Southeast Asia. In a pooled multivariable analysis, travel destination, age, overnight hospitalisation abroad, TD, and use of fluoroquinolones were independently associated with increased ESBL-PE colonization rates.

**Conlusions:**

Even in areas with relatively low risk of colonization, antimicrobials clearly predispose to colonization with ESBL-PE. Travellers to Africa should be cautioned against unnecessary use of antibiotics.

**Electronic supplementary material:**

The online version of this article (10.1186/s12879-018-3245-z) contains supplementary material, which is available to authorized users.

## Background

Every third traveller from industrialised countries that visits developing regions becomes colonized by extended-spectrum beta-lactamase-producing *Enterobacteriaceae* (ESBL-PE) [1–11]. While rates as high as 88% have been reported for travellers to South Asia [4–8, 10, 11] and 69% to Southeast Asia [4, 6–8, 10, 11], considerably lower risks have been detected among travellers to the African continent, varying between 12 and 45% [3, 4, 6–8, 10, 11].

Data on colonization among visitors to various African countries or subregions remain scarce, as the vast majority of prospective studies report acquisition rates either for the whole continent [1, 9] or only part of the subregions [3, 4, 6, 8–10, 12, 13]; a few investigations provide data on a number of individual countries [7, 11]. Travel destination, antibiotic use, age, and travellers’ diarrhoea (TD) have been identified as major risk factors. Several other additional factors have been shown in single studies: type of travel, meal location and consumption of certain food products, such as ice cream and pastries [1, 3–7, 9–11, 14]. While these factors all predispose to ESBL-PE colonization in general, studies presenting colonization risk factors by individual geographic areas are few [5, 11], and none have focused exclusively on Africa. As the continent attracts increasing numbers of travellers [15], we decided to review the data published and pool subregion-derived findings of our three earlier investigations [4 6, 10]. Combining these data with subregional carriage rates from two recent studies [7, 11], our paper offers an insight into the subregion-related colonization risk of travellers to Africa.

## Methods

### Study design, volunteers and samples

To assess the colonization rates of ESBL-PE in Africa, we combined the data of travellers to Africa from three large studies:

1. Finnish study by Kantele et al. [6]. The volunteers travelled in 2009–2010; 196 of the 430 (45.6%) travelled in Africa. Faecal samples were used for analyses.
2. Dutch study I by Paltansing et al. [4]. The volunteers travelled in 2011; 103 of 338 (30.5%) travelled in Africa. Rectal swabs were used for analyses.
3. Dutch study II by Reuland et al. [10]. The volunteers travelled in 2012–2013 (of the travellers reviewed here, all but one travelled in 2012); 97 of 418 (23.2%) travelled in Africa; 63 (64.9%) faecal samples and 34 (35.1%) rectal swabs were analysed. In the original article, the authors report that the colonization rates were similar regardless of sample technique.

For all three prospective studies, the volunteers provided both pre- and post-travel stool samples/rectal swabs. Of the 14 (3.5%) travellers with pre-travel samples positive for ESBL-PE, three had the same strain detected in post-travel samples. In six volunteers, the post-travel sample was negative; these were included in the ESBL-PE(−) group. The five that contracted a different type of ESBL-PE during travel were included in the ESBL-PE(+) group. Travellers who contracted a new ESBL-PE strain during travel constituted the ESBL-PE(+) group, while all others belonged to the ESBL-PE(−) group. The following information was available from all three studies in comparable format: travel itinerary, travel duration, travel dates, age, sex, antimicrobial usage, occurrence of TD, and possible hospitalisation abroad (overnight stay or more).

In all studies, written informed consent was obtained from all participants and the Ethics Committees in the respective organisations approved the study protocols.

### Collection of stool samples and identification of ESBL-PE strains

We have described earlier in detail the approaches to collection of samples (stools or swabs) and methods used for identification of ESBL-PE and carbapenemase-producing *Enterobacteriaceae* (CPE) [4, 6, 10].

### Definition of TD and geographical subregions

For the purpose of the present study, TD was defined as three or more loose or liquid stools per day. Geographical subregions in Africa were defined according to the United Nations [16]: Southern Africa, Western Africa, Middle Africa, Eastern Africa, and Northern Africa. Travellers visiting more than one subregion in Africa were categorised on the basis of longest stay.

### Statistical analyses

Statistical analyses were carried out with SPSS software version 24 (IBM Corp, Armonk, NY) and Stata version 15.1 (StataCorp. College Station, TX). Binomial regression model was used to obtain profile likelihood confidence intervals for the proportions of travellers with given risk factors and positive for ESBL-PE. The chi-square test, Fisher’s exact test or binary logistic regression analysis were used to compare categorical variables when applicable. Binary logistic regression was used with continuous variables. Variables with a *p*-value < 0.2 in the univariate analysis for ESBL-PE colonization were subjected to multivariable analysis together with doxycycline as antimalarial, gender and duration of travel in days. The shape of the form for travel duration and age were assessed by cubic splines and appeared log-linear. The interaction between variables of interest and studies was assessed. The final model was built using binary logistic regression analysis with a stepwise backward selection of variables by Akaike Information Criteria (AIC). Factors with 95% confidence intervals ranging only either above or below 1 were considered significant. The three studies pooled in the present paper [4, 6, 10] and the two others [7, 11] used for comparisons were all brought together to produce a forest plot analysis. Heterogeneity between studies in forest plot was measured with I^2^; values above 75% were considered high, 25–75% moderate, and below 25%. For our pooled data, the interaction between studies and geographical subregions was analysed in the multivariable model.

## Results

### Demographic data, background characteristics, and occurrence of TD

Demographic data on travellers are presented in Table 1. Of the 396 travellers included in this study, 237 (59.8%) were women. The median age was 36 years (IQR 27–53) and the median duration of travel 19 days (IQR 14–25). One fourth of the travellers (*n* = 105; 26.5%) had visited more than one country in Africa. The majority of the travellers visited either Western (27.8%) or Eastern (46.7%) Africa. Twenty-three (5.8%) had visited more than one subregion in Africa. In addition to Africa (or Europe *en route* to Africa), two volunteers (0.5%) had visited Jordania and two (0.5%) United Arab Emirates.

**Table 1:** Demographics and risk factors of ESBL-PE acquisition in pooled data on 396 travellers from Finland and the Netherlands

				Univariate analysis			Multivariable analysis		
Characteristic	total n (% of all)	ESBL-PE(+) n (%)	95% CI (%)^a^	P	OR	95% CI	P	AOR	95% CI
Total	396 (100.0)	61 (15.4)							
Gender									
Male	159 (40.2)	24 (15.1)	10.1–21.2		1.0				
Female	237 (59.8)	37 (15.6)	11.4–20.6	0.889	1.0	0.6–1.8			
Age^b^									
Age, median, years	36 (IQR 27–53)	38.5 (IQR 25.5–55.5)		0.071	1.0	1.0–1-0	0.002	1.0	1.0–1.1
Study									
Kantele et al. [6]	196 (49.5)	25 (12.8)	8.6–17.9		1.0			1.0	
Paltansing et al. [4]	103 (26.0)	29 (28.2)	20.1–37.3	0.001	2.7	1.5–4.9	0.303	3.7	0.3–50.0
Reuland et al. [10]	97 (24.5)	7 (7.2)	3.2–13.5	0.158	0.5	0.2–1.3	0.962	1.1	0.1–25.1
Sampling method									
Rectal swab	137 (34.6)	32 (23.4)	16.8–30.9	0.001	2.4	1.4–4.2	0.648	1.5	0.3–7.8
Stool sample	259 (65.4)	29 (11.2)	7.7–15.4		1.0				
Year of travel^b^									
(year of travel as a continuous variable)				0.909	1.0	0.8–1.2	0.609	0.8	0.3–2.1
2009	122 (30.8)	17 (13.9)	8.6–20.8						
2010	74 (18.7)	8 (10.8)	5.1–19.2						
2011	103 (26.0)	29 (28.2)	20.1–37.3						
2012–2013	97 (24.5)	7 (7.2)	3.2–13.5						
Destination subregion									
Southern Africa	58 (14.6)	4 (6.9)	2.2–15.3		1.0			1.0	
Northern Africa	28 (7.1)	12 (42.9)	25.8–61.2	< 0.001	10.1	2.9–35.8	0.001	12.4	3.1–57.3
Middle Africa	15 (3.8)	4 (26.7)	9.2–51.5	0.042	4.9	1.1–22.7	0.056	5.6	0.9–33.6
Eastern Africa	185 (46.7)	30 (16.2)	11.4–22.0	0.084	2.6	0.9–7.8	0.058	3.1	1.1–11.2
Western Africa	110 (27.8)	11 (10.0)	5.3–16.5	0.505	1.5	0.5–4.9	0.528	1.5	0.4–6.2
Antibiotics									
no AB	352 (88.9)	44 (12.5)	9.3–16.2		1.0				
AB	44 (11.1)	17 (38.6)	25.2–53.4	< 0.001	4.4	2.2–8.7			
AB: beta-lactams									
No	388 (98.0)	57 (14.7)	11.4–18.4		1.0			1.0	
Yes	8 (2.0)	4 (50.0)	19.1–80.9	0.022	5.8	1.4–23.9	0.118	3.4	0.5–21.9
AB: fluoroquinolones									
No	371 (93.7)	51 (13.7)	10.5–17.5		1.0			1.0	
Yes	25 (6.3)	10 (40.0)	22–5-59.5	0.002	4.2	1.8–9.8	0.005	4.7	1.5–13.9
AB others (other than beta-lactams or FQ)/unknown									
No	382 (96.5)	56 (14.7)	11.4–18.4		1.0			1.0	
Yes	14 (3.5)	5 (35.7)	14.6–61.7	0.048	3.2	1.0–10.0	0.059	3.8	0.9–14.6
Doxycycline as antimalarial									
No	362 (91.4)	56 (15.5)	12.0–19.4		1.0				
Yes	34 (8.6)	5 (14.7)	5.5–29.0	0.906	0.9	0.4–2.5			
Travellers’ diarrhoea (TD)									
no TD	243 (61.4)	30 (12.3)	8.6–16.9		1.0				
TD	153 (38.6)	31 (20.3)	14.4–27.1	0.034	1.8	1.0–3.1	0.033	2.1	1.1–4.1
Overnight hospitalisation abroad (information missing *n* = 1)									
No	389 (98.5)	57 (14.7)	11.4–18.4		1.0				
Yes	6 (1.5)	4 (66.7)	28.1–100	0.006	11.6	2.1–65.1	0.004	16.5	2.5–140.5
Duration of travel, (information missing *n* = 1)^b^									
Median, days	19 (IQR 14–25)	18 (IQR 15–23)		0.828	1.0	1.0–1.0	0.276	1.0	1.0–1.0

^a^95% confidence intervals are profile likelihood confidence intervals for proportion of ESBL(+) with given risk factor

^b^studied as a continuous variable in statistical analysis

TD rates were lowest in Southern Africa (15/58; 25.9%); in other areas 37.8–46.7% of travellers contracted TD (Table 2.). Of the 44 (11.1% of all travellers) courses of antibiotics, 30 (68.2%) were taken for TD. Eight (2.0% of all travellers) used beta-lactam antibiotics and 25 (6.3%) used fluoroquinolone antibiotics during travel.

**Table 2:** ESBL-PE colonization rates, occurrence of TD and antibiotic use in the pooled data on 396 travellers from Finland and the Netherlands in relation to geographical subregion visited

	All	Northern Africa	Middle Africa	Eastern Africa	Western Africa	Southern Africa
	n (%)	n (%)	n (%)	n (%)	n (%)	n (%)
Kantele et al. (Finnish study) [6]						
total no. of travellers to subregion (% of all)	196	3 (1.5)	4 (2.0)	86 (43.9)	78 (39.8)	25 (12.8)
ESBL-PE (+)	25 (12.8)	2 (66.7)	1 (25.0)	14 (16.3)	5 (6.4)	3 (12.0)
AB	34 (17.3)	1 (33.3)	1 (25.0)	13 (15.1)	15 (19.2)	4 (16.2)
TD	71 (36.2)	1 (33.3)	1 (25.0)	34 (39.5)	29 (37.2)	6 (24.0)
Paltansing et al. (Dutch study I) [4]						
total no. of travellers to subregion (% of all)	103	13 (12.6)	7 (6.8)	54 (52.4)	12 (11.7)	17 (16.5)
ESBL-PE (+)	29 (28.2)	7 (53.8)	3 (42.9)	14 (25.9)	4 (33.3)	1 (5.9)
AB	8 (7.8)	2 (15.4)	1 (14.3)	3 (5.6)	2 (16.7)	0 (0.0)
TD	39 (37.9)	7 (53.8)	4 (57.1)	19 (35.2)	5 (41.7)	4 (23.5)
Reuland et al. (Dutch study II) [10]						
total no. of travellers to subregion (% of all)	97	12 (12.4)	4 (4.1)	45 (46.4)	20 (20.6)	16 (16.5)
ESBL-PE (+)	7 (7.2)	3 (25.0)	0 (0.0)	2 (4.4)	2 (10.0)	0 (0.0)
AB	2 (2.1)	0 (0.0)	0 (0.0)	2(4.4)	1 (16.7)	0 (0.0)
TD	43 (44.3)	5 (41.7)	2 (50.0)	17 (37.8)	14 (70.0)	5 (31.3)
Combined total of the three studies						
total no. of travellers to subregion (% of all)	396	28 (7.1)	15 (3.8)	185 (46.7)	110 (27.8)	58 (14.6)
ESBL-PE (+)	61 (15.4)	12 (42.9)	4 (26.7)	30 (16.2)	11 (10.0)	4 (6.9)
AB	45 (11.4)	3 (10.7)	2 (13.3)	18 (9.7)	18 (16.4)	4 (6.9)
TD	153 (38.6)	13 (46.4)	7 (46.7)	70 (37.8)	48 (43.6)	15 (25.9)

*AB* antibiotic use, *TD* travellers’ diarrhoea

### ESBL-PE acquisition rates by subregion in the pooled data

**Fig. 1:**
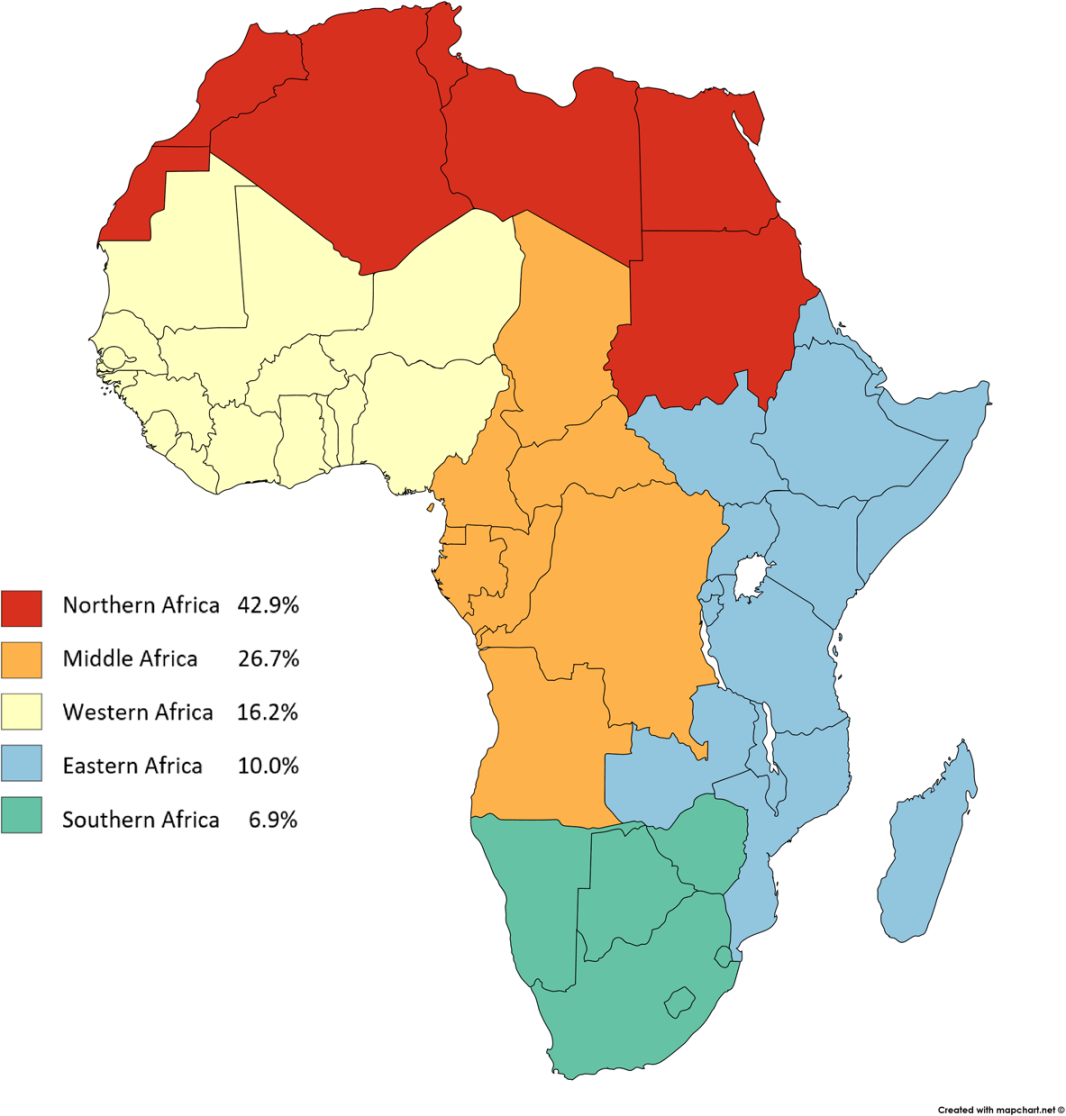
ESBL-PE acquisition rates in five African subregions; joint data on 396 travellers from Finland and the Netherlands. (Created with Mapchart.net)

In the pooled data (Table 2, Fig. 1), 61 (15.4%) travellers became colonized by ESBL-PE; one Dutch traveller to Egypt became colonized by CPE. The highest ESBL-PE colonization rates were seen among travellers to Northern Africa (12/28; 42.9%), followed by Middle Africa (4/15; 26.7%) and Eastern Africa (30/185; 16.2%). Of travellers to Western and Southern Africa, 10.0% (11/110) and 6.9% (4/58), respectively, acquired ESBL-PE (Tables 1 and 2).

Of the nine countries with more than 15 visitors (Table 3), the highest ESBL-PE acquisition rates were seen among travellers to Egypt (12/17; 70.6%), Ghana (6/23; 26.1%), Tanzania (14/81; 17.3%), Uganda (4/26; 15.4%) and Kenya (12/82; 14.6%). As for the lowest colonization rates, of the 26 travellers to Senegal, three (11.5%) became colonized by ESBL-PE, in South Africa, 5/49 (10.2%) became colonized and in the Gambia the rate was only 3.4% (2/58); none of the 21 visitors to Namibia acquired ESBL-PE.

**Table 3:** ESBL-PE colonization rates from our pooled data of 396 travellers by country visited presented with the respective figures from studies by Ruppé et al. [7] and Arcilla et al. [11]

Country	Data pooled from three studies^a^: Total number of travellers (% of all visitors to Africa)	Data pooled from three studies^a^: ESBL-PE (+) cases/all visitors to country (%)	Data published by Ruppé et al.^b^ ESBL-PE (+) cases/all visitors to country (%)	Data published by Arcilla et al.^c^ ESBL-PE (+) cases/all visitors to country (%)
Northern Africa				
Egypt	17 (4.3)	12/17 (70.6)	–	24/30 (80.0)
Morocco	10 (2.5)	1/10 (10.0)	–	8/36 (22.2)
Tunisia	3 (0.8)	0/3 (0)	–	–
Middle Africa				
Cameroon	7 (1.8)	1/7 (14.3)	13/24 (54.2)	–
Central African Republic	–	–	0/1 (0.0)	–
Democratic Republic of Congo	8 (2.0)	3/8 (37.5)	–	–
Republic of Congo	6 (1.5)	2/6 (33.3)	8/13 (61.5)	–
Gabon	1 (0.3)	0/1 (0)	2/3 (66.7)	–
Sao Tome and Principe	–	–	0/1 (0.0)	–
Eastern Africa				
Djibouti	1 (0.3)	1/1 (100.0)	–	–
Ethiopia	14 (3.5)	2/14 (14.3)	2/4 (50.0)	–
Kenya	82 (20.7)	12/82 (14.6)	4/6 (66.7)	10/30 (33.3)
Madagascar	3 (0.8)	1/3 (33.3)	4/7 (57.1)	–
Malawi	14 (3.5)	2/14 (14.3)	–	–
Mauritius	1 (0.3)	1/1 (100.0)	–	–
Mozambique	8 (2.0)	1/8 (12.5)	0/1 (0.0)	–
Rwanda	5 (1.3)	0/5 (0)	–	–
Tanzania	81 (20.5)	14/81 (17.3)	7/11 (63.6)	14/57 (24.6)
Uganda	26 (6.6)	4/26 (15.4)	–	12/27 (44.4)
Western Africa				
Benin	13 (3.3)	1/13 (7.7)	4/11 (36.4)	–
Burkina Faso	2 (0.5)	0/2 (0)	4/8 (50.0)	–
Cote d Ivoire	1 (0.3)	0/1 (0)	8/17 (47.1)	–
Gambia	58 (14.6)	2/58 (3.4)	–	8/49 (16.3)
Ghana	23 (5.8)	6/23 (26.1)	1/1 (100.0)	8/20 (40.0)
Guinea Bissau	1 (0.3)	0/1 (0)	0/3 (0.0)	–
Liberia	3 (0.8)	0/3 (0)	–	–
Mali	4 (1.0)	1/4 (25.0)	1/5 (20.0)	–
Nigeria	7 (1.8)	1/7 (14.3)	1/1 (100.0)	–
Senegal	26 (6.6)	3/26 (11.5)	17/45 (37.8)	–
Sierra Leone	3 (0.8)	1/3 (33.3)	–	–
Togo	6 (1.5)	2/6 (33.3)	9/12 (75.0)	–
Southern Africa				
Angola	1 (0.3)	0/1 (0)	1/3 (33.3)	–
Botswana	10 (2.5)	1/10 (10.0)	–	–
Lesotho	2 (0.5)	1/2 (50.0)	–	–
Namibia	21 (5.3)	0/21 (0)	–	–
South Africa	49 (12.4)	5/49 (10.2)	0/1 (0.0)	3/66 (4.5)
Swaziland	8 (2.0)	2/8 (25.0)	–	–
Zambia	13 (3.3)	1/13 (7.7)	–	–
Zimbabwe	7 (1.8)	1/7 (14.3)	–	–

There were no travellers to Burundi, Cape Verde, Chad, Equatorial Guinea, Eritrea, Guinea, Libya, Mauritania, Niger, Reunion, Seychelles, Somalia, South Sudan, and Sudan

^a^The same traveller may have visited several countries

^b^Only travellers that had visited only one country

^c^The colonization rates of all individual countries visited were not published

### Results of the multivariable analysis for risk factors of ESBL-PE colonization in Africa

The initial univariate analysis (Table 1) detected the following factors with *p* < 0.2: age, original study, sampling technique, subregion in Africa, use of fluoroquinolones, beta-lactams or other AB/regimen not known, TD, and overnight hospitalisation abroad. When all of these factors, together with gender, duration of travel, and use of doxycycline as antimalarial were subjected to multivariable analysis, the following were found to be independently associated with increased risk: travel to Northern Africa, overnight hospitalisation abroad, age, TD and use of fluoroquinolones (Table 1).

### Results of meta-analysis of five studies

**Fig. 2:**
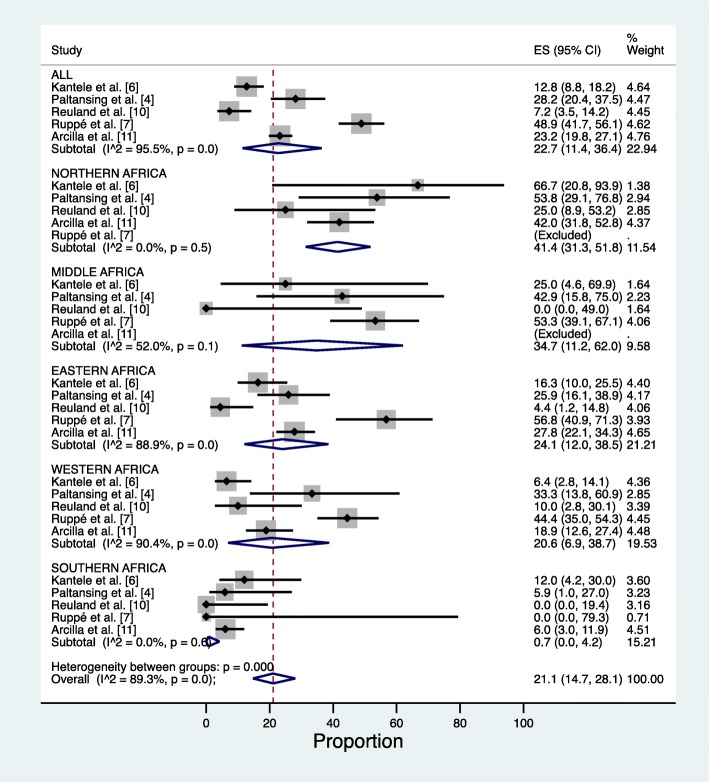
Forest plots of ESBL-PE acquisition rates from five studies in relation to geographical subregions. Excluded = no travellers to subregion in study

Table 4 and forest plot analysis (Fig. 2) show the ESBL-PE colonization rates from the three studies pooled [4, 6, 10], together with investigations by Ruppé et al. and Arcilla et al. [7, 11] in relation to geographical subregion visited. For Southern and Northern Africa, heterogeneity between the five studies appeared low (I^2^ = 0.0 and 0.0%, respectively), for Middle Africa moderate (I^2^ 52.0%), and Eastern and Western Africa high (I^2^ 88.9 and 90.4%, respectively). In the multivariable regression model of our pooled data, the interaction between subregions and the three studies was not found significant at 5% significance level.

**Table 4:** ESBL-PE colonization rates from the five studies [4, 6, 7, 10, 11] in relation to geographical subregion visited

	All	Northern Africa	Middle Africa	Eastern Africa	Western Africa	Southern Africa
Kantele et al. [6] (Finnish study) 2009–2010						
ESBL-PE (+) among travellers n (% of all visitors to subregion)	25/196 (12.8)	2/3 (66.7)	1/4 (25.0)	14/86 (16.3)	5/78 (6.4)	3/25 (12.0)
Paltansing et al. [4] (Dutch study I) 2011						
ESBL-PE (+) among travellers n (% of all visitors to subregion)	29/103 (28.2)	7/13 (53.8)	3/7 (42.9)	14/54 (25.9)	4/12 (33.3)	1/17 (5.9)
Reuland et al. [10] (Dutch study II) 2012–2013						
ESBL-PE (+) among travellers n (% of all visitors to subregion)	7/97 (7.2)	3/12 (25.0)	0/4 (0.0)	2/45 (4.4)	2/20 (10.0)	0/16 (0.0)
Ruppé et al. [7]2012–2013 (data on travellers visiting only one country)						
ESBL-PE (+) among travellers n (% of all visitors to subregion)	89/182 (48.9)	N/A	24/45 (53.3)	21/37 (56.8)	44/99 (44.4)	0/1 (0)
Arcilla et al. [11]2012–2013						
ESBL-PE (+) among travellers n (% of all visitors to subregion)	118/508 (23.2)	34/81 (42.0)	N/A	57/205 (27.8)	20/106 (18.9)	7/116 (6.0)
Combined total: ESBL-PE colonization rates	268/1086 (24.7)	46/109 (42.2)	28/60 (46.7)	108/427 (25.3)	75/315 (23.8)	11/175 (6.3)

## Discussion

Africa is a continent with increasing numbers of travellers [15]. When pooling subregion-/country-specific data from three traveller studies [4, 6, 10], we found the risk of contracting ESBL-PE to vary significantly between the various parts of Africa. In addition, comparing our joint data with two recent large reports [7, 11] providing subregion- and country-specific data enabled us to investigate the current subregion- and country-specific knowledge about ESBL-PE acquisition by travellers to Africa.

### ESBL-PE colonization rates in northern Africa

Our pooled data showed the highest acquisition rates (12/28; 42.9%) among visitors to Northern Africa, which accords with the results from the study by Arcilla et al. (42.0%) [11]; Ruppé et al. [7] did not report visitors to this subregion. Similar (43–44%) rates have been reported among Swedish travellers [3, 12]. Visitors to Egypt appear to be at particularly high risk; 70.6% (12/17) of our subjects, 80.0% (23/40) of those in a study by Arcilla et al., and 50% (19/38) of those in another by Tham et al. became colonized [11, 14]. Moreover, all 12 travellers colonized by ESBL-PE in Northern Africa had visited Egypt. It is noteworthy that these proportions are as high as those among travellers to India/South Asia in various investigations [4–8, 11]. Bassyouni et al. reported carriage rates as low as 21% among healthcare workers in Egypt [17].

### ESBL-PE colonization rates in middle Africa

To our knowledge, only one previous study has reported ESBL-PE acquisition rates among visitors to Middle Africa; Ruppé et al. [7] found 53.3% (24/45) of travellers to be colonized. In our pooled data, colonization rates in Middle Africa ranked second (4/15; 26.7%) among the subregions. In nonclinical samples obtained from local populations, carriage rates as high as 59% have been shown among healthy children in the Central African Republic [18], and 44–57% among inpatient carers, hospital workers and their household members in Cameroon [19].

### ESBL-PE rates in eastern Africa

Colonization rates among travellers to Eastern Africa (30/185; 16.2%) were lower than those reported by Arcilla et al. (57/205; 27.8%) [11], Lubbert et al. (12/47; 25.5%) [8], and Ruppé et al. (17/29; 56.8%,) [7]. Our moderate colonization rates are supported by findings among local populations: ESBL-PE carriage rates between 11.6 and 16.5% have been reported for healthy community children in Tanzania, [20, 21] and 5.3% for locals in Uganda, [22].

### ESBL-PE colonization rates in western Africa

ESBL-PE acquisition rates in Western Africa appear moderately low, but the results differ between studies: our pooled data showed proportions (11/110; 10.0%) close to those presented by Arcilla et al. (20/106; 18.9%) [11], while higher rates have been found by Ruppé et al. (44/99; 44.4%) [7] and Lubbert et al. (5/12; 38.5%) [8] among German travellers to Western and Middle Africa. Moreover, in the research by Frickmann et al., 27.1% (13/48) of European military personnel with diarrhoea in Mali became colonized by ESBL-PE [23]. As for local populations, colonization rates of 22% have been reported for healthy volunteers in Burkina Faso [24] and 33% for healthy community children in Guinea-Bissau [25].

### ESBL-PE colonization rates in southern Africa

Our low rates in Southern Africa (4/58; 6.8%) accord with those found by Arcilla et al. (7/116; 6.0%) [11] and Lubbert et al. (2/18; 11%) [8]. In our pooled data, the vast majority had visited South Africa or Namibia. Consistent with the low ESBL-PE acquisition rates, one study exploring local populations in South Africa reported maternal faecal carriage rates of 4.4% in South Africa [26].

### Findings from multivariable analysis

#### Travellers’ diarrhoea

ESBL-PE acquisition rates among those who contracted TD during travel (31/153; 20.3%) were higher than among those without TD (30/243; 12.3%) (AOR 2.1; 95% CI 1.1–4.1). This was expected, since TD was identified as a risk factor in two of the three original studies [6, 10] and numerous others [1, 3, 7, 8, 11, 12].

#### Antimicrobial medication

Forty-four (11.1%) travellers had taken antimicrobial medications during travel. Of the Finns, 17.3% (34/196) took antibiotics while this proportion was 5.0% (10/200) among the Dutch. In multivariable analyses, fluoroquinolone antibiotics were an independent risk factor for ESBL-PE colonization (ESBL-PE(+) 40.0%; AOR 4.7; 95% CI 1.5–13.9). Other antibiotic groups did not reach statistical significance in the risk factor analysis, yet the numbers of travellers using each individual antibiotic type were small; eight had taken beta-lactams (ESBL-PE(+) 50.0%; AOR 3.4, CI 0.5–21.9) and 14 other antimicrobials (ESBL-PE(+) 35.7%; AOR 3.8 CI 0.9–14.6). Ruppé et al. found beta-lactam usage to predispose to colonization by ESBL-PE (20/25; 80%) [7].

Even though taken by 34 (8.6%) travellers as an antimalarial, doxycycline was not associated with increased ESBL-PE rates (ESBL-PE(+) 5/34; 14.7%; AOR 0.9, 95% CI 0.4–2.5). This finding accords with other studies [7, 11]. However, these data do not allow conclusions on the total impact of doxycycline on antimicrobial resistance, as these investigations only analysed the ESBL or CPE feature of the *Enterobacteriaceae*; the potential to select doxycycline-resistant strains in general or other types of multidrug-resistant bacteria was not explored. Indeed, we recently showed that fluoroquinolone intake predisposes selectively to colonization by fluoroquinolone-resistant bacteria [27]. Thus, the effect of doxycycline on other bacteria and travellers’ microbiota deserves further research.

#### Increasing age as risk factor

Increasing age proved an independent risk factor for ESBL-PE colonization in Africa. Only two earlier reports [3, 6] have described similar results, as opposed to several others [7, 8, 11]. Moreover, in one study conducted among returning travellers with diarrhoea, increasing age even appeared protective [28]. The role of age remains unclear. There may be other factors associated with increasing age, such as co-medications/comorbidities or altered immune response not covered in these studies that interfere with the analyses in either direction. As the risk of bacteraemic infections caused by resistant *Enterobacteriaceae* increases with age [29], the risk factors in the older age groups warrant further studies.

#### Overnight hospitalisation

In our joint data, overnight hospitalisation predisposed to colonization with ESBL-PE. Although numerous retrospective studies have shown high colonization rates by multiresistant bacteria among travellers hospitalized in high-prevalence countries [30–32], to our knowledge, this is the first study to actually show in a prospective setting hospitalisation abroad as a risk factor for ESBL-PE acquisition. In previous prospective traveller studies, overnight hospitalisations has either not been analysed separately from other health care contacts in the risk factor analyses [7, 11] or the proportion of travellers requiring a stay in hospital for treatment has been small or negligible (0–0.5%) [3, 8]. In our data, six (1.5% of all subjects) needed overnight hospitalisation.

#### Travel destination

In multivariable analysis, when compared to Southern Africa, travel to Northern Africa was associated with higher colonization rates. The rates presumably vary between subregions and countries according to the background prevalence of the local populations [33]. They may also depend on several other factors, such as local culture-related food production and preparation habits and hygienic conditions and, of course, whether the traveller contracts TD and takes antibiotics (see above).

#### Other risk factors

Even though multiresistant *Enterobacteriaceae* have become increasingly prevalent globally [33], colonization rates were not found to increase during the study period (2009–2013). Neither individual studies nor sampling techniques were found statistically significant factors in the multivariable analysis. Travel duration was not seen to be associated with increased risk in univariate or multivariable analysis. This may be explained by a proportion of travellers becoming colonized already on arrival and the carriage resolving while abroad (Professor Kantele, unpublished observation).

### Limitations of the study

As the data for the joint risk factor analysis were derived from three separate studies, some data had been collected in differing formats rendering the results incomparable. Moreover, although pooling served to increase the validity and precision of study results, the data remained insufficient in some occasions for analysis in any great detail: In Additional file 1: Table S1, we present the factors available from two out of three studies [4, 6]: purpose of travel, diet (omnivore or vegetarian), type of accommodation, use of medications (antidiarrhoeals, proton-pump inhibitors, and antiemetics) and contact with local health care (other than hospitalisation). The five investigations appeared heterogeneous in the forest plot analysis, however, in the multivariable analysis of the pooled data, the interaction between subregions and studies was not found statistically significant.

Information concerning mild gastrointestinal symptoms in the ‘no TD’ group was only available for the Finnish volunteers (48.8% of all ‘no TD’ cases). To pool the three studies, we had to define TD as three or more stools per 24 h; milder diarrhoea cases were categorised as ‘no TD’, although even mild TD also may predispose to ESBL-PE acquisition.

## Conclusions

ESBL-PE colonization rates in African subregions appear moderate, with the exception of Northern Africa, especially Egypt. Also on this continent, however, TD and antibiotic use increase the risk of individual travellers acquiring ESBL-PE.

## Additional file

Additional file 1: **Table S1.** Factors available in same format only from the studies of Kantele et al. [6] and Paltansing et al. [4] and not included in the pooled data of this report. (DOCX 16 kb)

## Abbreviations

AB: Antibiotic
ESBL: Extended-spectrum beta-lactamase
ESBL-PE: Extended-spectrum beta-lactamase-producing *Enterobacteriaceae*
TD: Travellers’ diarrhoea
