# Supplementary material for: Extended-spectrum beta-lactamase-producing Enterobacteriaceae (ESBL-PE) among travellers to Africa: destination-specific data pooled from three European prospective studies
Source: BMC Infect Dis. 2018 Jul 23;18:341. doi: 10.1186/s12879-018-3245-z (PMC6057027; doi:10.1186/s12879-018-3245-z)
Supplement: Supplementary file 1 — Table S1. Factors available in same format only from the studies of Kantele et al. [6] and Paltansing et al. [4] and not included in the pooled data of this report. (DOCX 16 kb) [file 12879_2018_3245_MOESM1_ESM.docx]

Table S1 Factors available in same format only from the studies of Kantele et al [6] and Paltansing et al [4] and not included in the pooled data of this report.

|  | **total**  **n (% of all)** | **ESBL-PE (+)**  **n (%)** | **ESBL-PE (-)**  **n (%)** | **P-value** | **Univariate OR** | **95% CI** |
| --- | --- | --- | --- | --- | --- | --- |
| **Antidiarrhoeal medication** (information missing: 97; 24.5%) | | | | | | |
| None | 244 (81.6) | 41 (16.8) | 203 (83.2) |  | 1.0 |  |
| Loperamide | 52 (17.4) | 12 (23.1) | 40 (76.9) | 0.286 | 1.5 | 0.7-3.1 |
| Other | 3 (1.0) | 1 (33.3) | 2 (66.7) | 0.464 | 2.5 | 0.2-27.9 |
| **PPI/antacid** (information missing: 98; 24.7%) | | | | | | |
| No | 277 (93.0) | 50 (18.1) | 227 (81.9) |  | 1.0 |  |
| PPI | 18 (6.0) | 4 (22.2) | 14 (77.8) | 0.658 | 1.3 | 0.4-4.1 |
| Other antacid | 3 (1.0) | 0 (0) | 3 (100.0) | 0.999 | n/a | n/a |
| **Purpose of travel** (information missing: 99; 25.0%) | | | | | | |
| Holiday | 216 (72.7) | 38 (17.6) | 178 (82.4) |  | 1.0 |  |
| Work/business | 37 (12.5) | 5 (13.5) | 32 (86.5) | 0.679 | 0.8 | 0.3-2.2 |
| Living abroad | 14 (4.7) | 3 (21.4) | 11 (78.6) | 0.612 | 1.4 | 0.4-5.3 |
| Studying/volunteering | 21 (7.1) | 7 (33.3) | 14 (66.6) | 0.057 | 2.6 | 1.0-6.7 |
| VFR | 9 (3.0) | 4 (44.4) | 5 (55.6) | 0.041 | 4.1 | 1.1-16.2 |
| **Antiemetics** (information missing: 98; 24.7%) | | | | | | |
| No | 293 (98.3) | 55 (18.8) | 238 (81.2) |  | 1.0 |  |
| Yes | 5 (1.7) | 2 (40.0) | 3 (60.0) | 0.244 | 2.9 | 0.5-17.7 |
| **Diet** (information missing: 164; 41.4%) | | | | | | |
| Omnivore | 214 (92.2) | 49 (22.9) | 165 (77.1) |  | 1.0 |  |
| Vegetarian | 18 (7.8) | 2 (11.1) | 16 (88.9) | 0.247 | 0.4 | 0.1-1.9 |
| **Health care contact** (information missing: 97; 24.5%) | | | | | | |
| No | 274 (91.6) | 50 (18.2) | 224 (81.8) |  | 1.0 |  |
| Yes | 25 (8.4) | 7 (28.0) | 18 (72.0) | 0.235 | 1.7 | 0.7-4.4 |
| **Accommodation**  (information missing: 105; 26.5%) | | | | | | |
| Hotel | 152 (52.2) | 23 (15.1) | 129 (84.9) |  | 1.0 |  |
| Guesthouse / lodge | 62 (21.3) | 13 (21.0) | 49 (79.0) | 0.302 | 1.5 | 0.7-3.2 |
| With locals / own home | 56 (19.2) | 10 (17.9) | 46 (82.1) | 0.634 | 1.2 | 0.5-2.8 |
| Other | 21 (7.2) | 7 (33.3) | 14 (66.7) | 0.045 | 2.8 | 1.0-7.7 |
